# Poly(vinyl alcohol)/Plant Extracts Films: Preparation, Surface Characterization and Antibacterial Studies against Gram Positive and Gram Negative Bacteria

**DOI:** 10.3390/ma15072493

**Published:** 2022-03-28

**Authors:** Mihaela Barbălată-Mândru, Diana Serbezeanu, Maria Butnaru, Cristina Mihaela Rîmbu, Alexandru Alin Enache, Magdalena Aflori

**Affiliations:** 1 “Petru Poni” Institute of Macromolecular Chemistry, 41A Aleea Gr. GhicaVoda, 700487 Iasi, Romania; mandru.mihaela@icmpp.ro (M.B.-M.); mariabutnaru@yahoo.com (M.B.); 2 Department of Biomedical Sciences, “Grigore T. Popa” University of Medicine and Pharmacy, 9-13, Kogalniceanu Street, 700115 Iasi, Romania; 3 Department of Public Health, Faculty of Veterinary Medicine, Iasi University of Life Sciences (IULS), Mihail Sadoveanu Alley no. 8, 700490 Iasi, Romania; crimbu@uaiasi.ro; 4 S.C. Apel Laser S.R.L., 25, Vanatorilor Street, Mogosoaia, 077135 Ilfov, Romania; alin.enache@apellaser.ro

**Keywords:** poly(vinyl alcohol), extract of *Lavandula angustifolia*, *Mentha piperita*, *Cannabis sativa* L., *Verbena officinalis*, *Salvia officinalis folium*, cytotoxicity, antibacterial activity

## Abstract

In this study, we aim to obtain biomaterials with antibacterial properties by combining poly(vinyl alcohol) with the extracts obtained from various selected plants from Romania. Natural herbal extracts of freshly picked flowers of the lavender plant (*Lavandula angustifolia*) and leaves of the peppermint plant (*Mentha piperita),* hemp plant (*Cannabis sativa* L.), verbena plant *(Verbena officinalis)* and sage plant *(Salvia officinalis folium)* were selected after an intensive analyzing of diverse medicinal plants often used as antibacterial and healing agents from the country flora. The plant extracts were characterized by different methods such as totals of phenols and flavonoids content and UV-is spectroscopy. The highest amounts of the total phenolic and flavonoid contents, respectively, were recorded for *Salvia officinalis.* Moreover, the obtained films of poly(vinyl alcohol) (PVA) loaded with plant extracts were studied concerning the surface properties and their antibacterial or cytotoxicity activity. The Attenuated Total Reflection Fourier Transform Infrared analysis described the successfully incorporation of each plant extract in the poly(vinyl alcohol) matrix, while the profilometry demonstrated the enhanced surface properties. The results showed that the plant extracts conferred significant antibacterial effects to films toward *Staphylococcus aureus* and *Escherichia coli* and are not toxic against fibroblastic cells from the rabbit.

## 1. Introduction

The progress of different materials with antibacterial properties appertains to a research area that is always in intensively expanding. Moreover, different materials have the potential to prevent the proliferation of strains, providing protection against a lot of pathogenic microorganisms such as *Escherichia coli* and *Staphylococcus aureus,* two species of Gram-positive and Gram-negative strains responsible for diseases such as skin and strong bloodstream infections [1]. Some statistics from the Broad Institute showed that these bacteria were responsible for clinical infections in proportions of 18.8% (*S. aureus*) and 17.3 % (*E. Coli*) [2]. Because *S. aureus* and *E. coli* are bacteria resistant to a lot of antibiotic therapy, major concerns were raised in the public health field regarding the necessity to develop other new antibacterial compounds. For this reason, the current interest of science is focused on biodegradable polymers to be used for this purpose [3,4,5]. Poly(vinyl alcohol) (PVA) is one of the best known environmentally friendly polymers and is obtained in a hydrolysis reaction of poly(vinyl acetate) [6]. The polymer is characterized by a lot of significant features such as water solubility, non-toxicity, chemical resistance, biocompatibility and biodegradability [7,8,9]. One of its important features is the hydrophilicity. The hydroxyl groups from this structure support the good film-forming ability and intermolecular hydrogen bonding formation [6,10]. For all the mentioned features, the polyvinyl alcohol polymer presented a lot of interest for the researchers. Moreover, the antibacterial compounds, such as extracted plants, are used to increase the capacity of biopolymers to kill microorganisms from the surface of the used materials [11,12,13]. The composition of each extract varies depending on the plant and what extraction process is used [12,13,14]. The medicinal plants contain several phytochemicals such as phenols acids, flavonoids, alkaloids, terpenoids and tannins who possess a lot of bioactive properties and, among them, also different antibacterial effects. Thus, the antibacterial effect of these plants secondary metabolites is associated to -OH group(s) attached to the phenol ring [15]. The phenolic acids represent a family of organic compounds that have a phenolic ring and a carboxylic acid group in their structure. The antibacterial effect is related to the number of -OH and methoxy (-OCH3) groups present. Due to their character of weak acids, they have the possibility to diffuse through the bacterial membrane, leading to cell death after the cytoplasm acidifying [16]. Furthermore, the flavonoids, a huge group of bioactive substance, possess antibacterial activity against a wide array of microorganisms. Their antibacterial effect is probably due to the formation of some complexes with the bacterial cell wall and disrupts the cell membranes [17]. Regarding the terpenes, they are some polymers of isoprene whose antibacterial effect was related to the presence of hydroxyl groups carbonylation of terpenoids or their lipophilicity/hydrophobicity, causing an alteration to the photorespiratory pathway [18]. Furthermore, another complex of polymers, tannins, exerts its antibacterial effect by complexing with proteins through both covalent and non-covalent interactions [17]. Phytochemicals that are characterized by diverse mechanisms of action, and chemical structures are very attractive for therapeutic tools. The prepared manuscript uses five different herbs: *Lavandula angustifolia*, *Mentha piperita,* *Cannabis sativa* L., *Verbena officinalis* and *Salvia officinalis folium*. As described in literature, these plants were chosen because of their known antibacterial activity [11,13,19,20].

*Lavandula angustifolia* (*Lamiaceae* family) is a perennial, aromatic and medicinal plant [12,13] with a lot of benefits to human health. Due to the presence of its components, the obtained lavender extract exerts different effects such as anti-inflammatory or antibacterial [21]. Externally, it can be used for wounds and superficial burns or oral hygiene [13].

*Mentha piperita* (*Lamiaceae* family) is a plant cultivated worldwide [20]. The largest exporters of peppermint on the global market are the U.S.A. and Great Britain [22]. Previous studies of peppermint oils showed their composition and their therapeutic properties [22,23]. They contain menthol, menthyl acetate, menthone, linalool, carvone, limonene, etc. [23,24]. These properties have ensured a lot of applications such as in medicine and the food industry [20,21,22].

The most common plant, used from ancient time for many ailments, is the hemp plant (*Cannabis sativa* L.) from the *Cannabaceae* family [25,26]. *Cannabis sativa* L. contains numerous active compounds. A lot of them belong to the primary metabolites, for example steroids, amino acids, fatty acids and the cannabinoids, flavonoids, lignans, terpenoids and alkaloids, which appertain to the secondary metabolites [27]. The therapeutic proprieties for cancer or various other diseases are intensely studied, but few studies are using hemp plant as an antibacterial agent [25,26,27].

*Verbena officinalis* L. (*Verbenaceae* family) is a perennial herbaceous herb that blossoms from April until October [28]. It contains different compounds such as terpenoids, steroids, tannins, etc. [29]. The herb *Verbena officinalis* has large repute in fighting infections [30]. Its history reveals a number of large medicinal aspects, such as a topical anti-inflammatory or antifungal agent [30].

*Salvia officinalis* L. (*Lamiaceae* family) is one of the most popular medicinal plants. Besides many others therapeutic properties, *S. officinalis* has a large spectrum with antibacterial activity [31,32]. It has very rich components of phenols (rosmarinic, ferulic and caffeic acid), flavonoids (luteolin, apigenin and naringenin) and, mainly, terpenoids (such as α- and β-thujone, camphor and 1,8-cineole) [11,32]. The preparations based on *Salvia officinalis* cn be used in different forms such as liquid extracts, powder forms or essential oils [32,33].

In this study, polymer films were prepared from poly(vinyl alcohol) (PVA) loaded with natural herbal extracts of freshly picked flowers of the lavender plant (*Lavandula angustifolia*), leaves of the peppermint plant (*Mentha piperita*), hemp plant (*Cannabis sativa* L.), verbena plant (*Verbena officinalis*) and sage plant (*Salvia officinalis folium*) in order to present their functionality as antibacterial materials with potential medical applications. To establish a wide range of properties for the compounds of the plant extracts, some studies were made such as the total phenols and flavonoids content and UV-is spectroscopy analysis. The obtained films of poly(vinyl alcohol) (PVA) loaded with plant extracts were studied concerning the modification of the surface properties with ATR-FTIR analysis, roughness or contact angle measurement. The antibacterial effects of films and their cytotoxicity were made in vitro. The results showed that the films loaded with plant extracts present significant antibacterial effects toward *Staphylococcus aureus ATCC 25923* and *Escherichia coli ATCC 25922)* and are not toxic.

## 2. Materials and Methods

### 2.1. Materials

Poly(vinyl alcohol) (Mw = 130,000 Da, 87–89% hydrolyzed) was purchased from Sigma-Aldrich (St. Louis, MO, USA). DMEM (Dulbecco's Modified Eagle Medium), Phosphate Buffered saline, 3-(4,5-dimethyl-2-thiazoyl)-2,5-diphenyl-2H-tetrazolium bromide (MTT), Fetal Bovine Serum, Penicillin/Streptomycin/Neomycin solution and solution in PBS (5 mg/mL) were purchased from Sigma-Aldrich Chemistry, Steinheim, Germany. Freshly picked flowers of *Lavandula angustifolia* and leaves of *Mentha piperita*, hemp plants (*Cannabis sativa* L.), *Verbena officinalis* and *Salvia officinalis folium* were purchased from different local markets such as Bioskin SRL and Agro Denmar SRL in Romania. The various samples were washed using distilled water and after that were allowed to dry at laboratory ambient temperature (23 ± 5 °C) [34]. All other reagents were used as received from commercial sources or were purified by standard methods.

### 2.2. Obtainment of Plant Extracts

An amount of dried buds or leaves plants were ground manually in a mortar. In order to achieve reproducible extraction yields, they were passed through a sieve with the sizes of mesh between 20 and 30 and the particle diameters varying over 0.60–0.85 mm. The obtained samples were kept until they were used in a sealed bag in a cold and dry place [35]. The extraction was carried out in a Soxhlet apparatus (Aldrich^®^ Soxhlet Extraction Apparatus, Z556203, St. Louis, MO, USA) for 6 h. A known amount (6 g) of dried samples was added in 150 mL of ethanol and was subjected to this extraction. The temperature was maintained at 60 °C during the extraction. After this, the solvent was evaporated under reduced pressure using rotary evaporator (Heidolph Instruments GmbH& CoKG, Schwabach, Germany) at 45 °C in order to obtain the crude ethanol extract. Then, they were stored at −20 °C until further use. Five different batches of diverse extract were prepared. The extraction yield was calculated for each plant extract according to the following:

(1) $$Yields\;\left(\%\right)=\frac{Wi-Wf}{Wi}*100$$

where Wi represents the weight of the used plant and Wf the weight of the obtained extract. Yields of 10 ± 1%, 13 ± 2%, 11.2 ± 1%, 10.5 ± 2% and 11.2 ± 2% were obtained for *Lavandula angustifolia*, *Salvia officinalis folium* [14], hemp extracts (*Cannabis sativa* L.), *Mentha piperita* and *Verbena officinalis,* respectively.

### 2.3. Films Preparation

**Figure 1:**
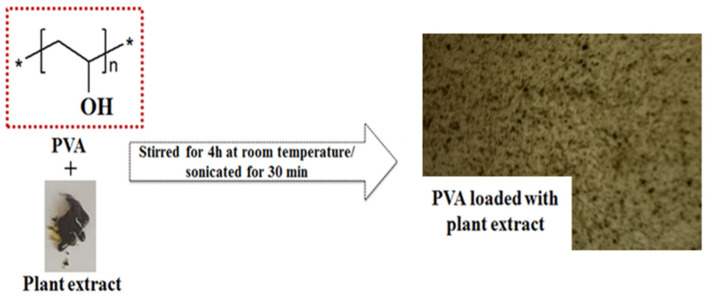
Schematic representation of PVA film loaded with plant extract.

The poly(vinyl alcohol) films with plant extracts were prepared by using the solvent-casting method. In this respect, 10% *w/v* of poly(vinyl alcohol) (PVA) was dissolved in distilled water and heated at 80 °C for 3 h, and the solution was equilibrated at room temperature overnight. Subsequently, 0.1% wt. of plant extracts (based on weight of PVA) was added into PVA solution. The mixture was stirred for 4 h at room temperature in order to achieve a good dispersion of the plant extract into the PVA matrix. The prepared solutions were sonicated for 30 min to remove the bubbles with an ultrasonic cleaner (Model FB11012, Fisherbrand, Loughborough, UK). Subsequently, the mixtures (15 mL) were cast in different Petri dishes with a diameter of 9 cm and dried at 50 °C for 2 h. The obtained films were stored at a constant temperature (25 °C) in a humidity chamber for at least 2 days before characterization tests. A representation of the film preparation is described in the Figure 1.

### 2.4. Determination of Total Polyphenol and Flavonoids Content

The total phenolic content (TPC) was made using some amount of plant extracts such as *Lavandula angustifolia*/*Verbena officinalis*/*Salvia officinalis*/*Hemp*/*Mentha piperita,* which were mixed with ethanol (2 mL) on a magnetic stirrer at 1500 rpm and room temperature. In this regard, the Folin-Ciocalteu (FC) method was adapted to determine the total content of phenolic compounds that was used [36]. The FC reagent was diluted with ethanol/distilled water in a ratio of 1:10 (*v/v*). To prepare the sodium carbonate solution (20%), the sodium carbonate (75 g/L) was dissolved with distilled water in a volumetric flask. Then, 1 mL or standard sample was mixed with 10 mL of diluted solution of FC reagent. After 6 min, 1.5 mL of sodium carbonate (20%) was added, and the mixture was kept in the dark a period of 90 min. At the end of this period, the absorbances were recorded at 760 nm with a UV-VIS spectrophotometer (SPECORD 21 Plus Analytik, Jena, Germany). Gallic acid was used to obtain a standard calibration curve (6–30 µg/mL). The results were calculated from the obtained calibration curve being expressed as milligrams of gallic acid equivalents per gram of dry weight (mg GAE /g extract). All experiments were performed in triplicate.

The colorimetric assay was used to determine the total flavonoid content described by Prundeanu et al. [37] with some minor modifications. Thus, 0.5 mL of each extract was mixed separately with 1.5 mL of ethanol (99.7–100% *v*/*v*), 0.1 mL of potassium acetate (1 M), 0.1 mL of AlCl_3_ (10%) and an amount of 2.8 mL of distilled water. The resulting mixture was incubated in the dark for 40 min. The absorbance of the mixture was recorded at 415 nm by using a UV-VIS spectrophotometer. Quercetin was used to construct the standard calibration curve (10–100 µg/mL). The results were expressed as milligrams of quercetin equivalents per gram of dry weight (mg QE/g extract) [37].

### 2.5. UV-VIS Spectra

Ultraviolet-visible (UV-VIS) spectra of extracts were recorded using a UV spectrophotometer (SPECORD 21 Plus Analytik, Jena, Germany). The interval for scans was in the spectral range of 200–500 nm. The test was repeated three times for each sample.

### 2.6. Fourier-Transform Infrared Spectroscopy Analysis

The infrared spectra of the studied films were obtained using a Bruker LUMOS—FTIR Microscope (BrukerOptik GmbH, Ettlingen, Germany) with the ATR reflection module (attenuated total reflection) and a diamond crystal at a single reflection 45° angle equipped with OPUS 8 software (Version 8, Ettlingen, Germany) for spectral processing. The surfaces samples were scanned in the 600–4000 cm^−1^ range. The plant extracts were recorded in a transmission module. All spectra were collected by cumulating 64 scans at a constant temperature of 25 °C.

### 2.7. Contact Angle and Surface Free Energy

In order to record the surface free energy of the samples, the static contact angles were measured with a static drop technique, which has a KSV CAM 101 goniometer equipped with a special optical system and a CCD camera connected to a computer. A drop of liquid (~1 μL) was placed on a specially prepared plate of substratum with a Hamilton syringe, and the images were immediately sent via the CCD camera to the computer for analysis. All the measurements were conducted in triplicate and the results were recorded as mean ± standard deviation. The angle formed between the liquid/solid interface and the liquid/vapor interface is the contact angle. Temperature and moisture were constant during the experiment (25 °C and 65%, respectively). The results of the measurements for each films and each testing liquid were recalculated by the Owens–Wendt–Rabel and Kaelble (OWRK) method [38] into total surface free energy (γ_sv_) with polar (γ^p^_sv_) and dispersive (γ^d^_sv_) components.

### 2.8. Profilometry Analysis

A stylus profiler Tencor Alpha-Step D-500 was used for studying the surface topography of the samples. The arithmetical mean surface roughness values (R_a_) were estimated from an average of five profiles (KLA Tencor Corporation, Milpitas, CA, USA). The measured heights of the steps have an accuracy ranging between 10 Å and 1.2 mm. The device measures the roughness parameters with a recording speed of 0.10 mm/s and a filtration interval of 0.060 mm. Five profiles were recorded for each sample and the surface roughness was measured in nm.

### 2.9. Raman Microscopy Analysis

Confocal images were recorded using an inVia™ confocal Raman microscope spectrometer (Renishaw plc, Gloucestershire, UK) equipped with a 633 nm excitation laser line and a Leica DM2700 microscope with 5×, 20×, 50× and 100× objectives and a Deep Depletion Renishaw CCD Centrus array detector (Renishaw plc, Gloucestershire, UK).

### 2.10. Mechanical Properties

The mechanical measurements of stress–strain were performed on an Instron apparatus (INSTRON model 3365; Universal Testing Machine, INSTRON, Norwood, MA, USA). A load cell of 500 N was used on dumbbell-shaped cut samples of 50 mm total length, 4 mm active area width and 8.5 mm gripped width. The thickness was measured for each cut mat with a digital micrometer. The tests were performed at an extension rate of 10 mm min^−1^ at room temperature (20–22 °C). For statistical significance, 3 specimens of each sample were tested, and average values of strength and elongation at break were determined.

### 2.11. Scanning Electron Microscopy (SEM)

The morphology of the surface was examined using a FEI Quanta 200 scanning electron microscope (FEI Company, Brno, Czech Republic) in a high-vacuum microscope chamber. The apparatus is equipped with an EDAX Si (Li) X-ray detector (FEI Company, Brno, Czech Republic) and Gatan Alto Cyrostage (FEI Company, Brno, Czech Republic) and uses an accelerating voltage of 20 k. The samples were mounted on different supports being observed under different degrees of magnifications. Each sample was analyzed by several measurements. 

### 2.12. Antibacterial Analysis

The antibacterial properties of the obtained PVA films loaded with plant extracts were evaluated by the Kirby–Bauer disk diffusion method [39]. *Staphylococcus aureus* ATCC 25923 (Gram-positive bacteria) (Thermo Fisher Scientific Inc., Dartford, UK) and *Escherichia coli* ATCC 25922 (Gram-negative bacteria) (Thermo Fisher Scientific Inc, Dartford, UK) were put in contact with the PVA film samples (6 mm). The protocol implied the preparation of a bacterial inoculum with 0.9% NaCl dilution and a turbidity of 0.5 on the McFarland scale (1.5 × 10^8^ bacterial cells/mL) for 24 h of cultured cells. The bacterial cultures were incorporated in a sterile Mueller–Hinton (Oxoid), melted and cooled to 45 °C. The PVA films loaded with plant extracts and paper discs impregnated with extracts (80 µL) were placed at a relatively equal distance between them onto one Petri dish with Mueller–Hinton agar, inoculated with bacteria suspensions. The plates were prepared in duplicate and incubated at 37 °C for 24 h. After the incubation, the area of the microbial inhibition zone for each sample was measured.

### 2.13. Cytotoxicity Assay 

#### 2.13.1. Cell Culture Conditions

The used cells in the biocompatibility analysis were the rabbit primary fibroblasts, stored at −86 °C. These cells were thawed by immersing the flask in a bath with heated water at 37 °C under gentle stirring in order to accelerate the process of the ice melting and uniformity of the suspension. After the complete melting of the ice, Dulbecco's Modified Eagle Medium (DMEM) culture medium with 10% fetal calf serum and 1% mixture of penicillin–streptomycin–neomycin antibiotics (all for in vitro use) was added to the thawed cells. Then, 24-well culture plates were seeded with a number of 10,000 cells/well, over which a fragment of our studied samples was added in inserts with porous membranes (0.4 µm) using the indirect contact system. Before viability assay, the PVA film and PVA film were loaded with a plant extracts disc of 6 mm in a hood with vertical sterile air flow and immersed in DMEM culture medium with 10% fetal calf serum and 1% mixture of penicillin–streptomycin–neomycin antibiotics. 

#### 2.13.2. MTT Assay

MTT assay is a method used to determine the activity of cellular metabolism by using tetrazolium salts as an oxidized substrate for mitochondrial dehydrogenases [40]. The principle of the method is based on the reduction of the yellow MTT compound into an insoluble, purple (formazan) product. Briefly, after incubation of the films loaded with plant extracts with the cells, the MTT solution (1 mg/mL) was added and it was incubated for 3 h in the dark, at 37 °C. Then, the formazan crystals were solubilized by adding 0.5 mL/wall of isopropanol, and after this a blue-violet coloration was achieved. The absorbance of the colored obtained solution was quantified by the spectrophotometer measuring at λ = 570 nm. The cell viability was normalized to that of rabbit primary fibroblasts cultured in the media with the negative control (without material).

## 3. Results and Discussion

### 3.1. ATR-FTIR Analysis

The objective of ATR-FTIR analysis was to investigate the registered peaks corresponding to the functional groups (OH-, C-O and C-H) indicating the presence of the phenols acid, terpenoids or flavonoids compounds and observing the influence of plant extracts on each PVA formulation (Table S1, See Supplementary Information).

Regarding the ISO Standard 3515, the identification of individual components of *L. angustifolia* are as follows: 1,8-cineole, 0–15%; limonene, 0–0.5%; trans-b-ocimene, 2–6%; cis-b-ocimene, 4–10%; 3-octanone, 0–2%; camphor, 0–0.5%; linalool, 25–38%; linalyl acetate, 25–45%; terpinen-4-ol, 2–6%; lavandulol minimum, 0.3%; lavandulyl acetate minimum, 2.0% and α-terpineol, 0–1%. Thus, the major constituents are linalool, linalyl acetate, terpinen-4-ol and camphor [13].

According to Beiji et al. [23], in fresh peppermint, 36 components were identified in total that accounting for 93.84% of the total volatile. Nonetheless, the main components generally reported of the *Mentha piperita* extracts were the phenolic acids, flavonoids, terpenoids (menthol and carvone) or terpenes (1,8-cineole, menthone, menthyl acetate, linalool, limonene and pinene), respectively.

Additionally, Elhendawy et al. [19] described the hemp (*Cannabis sativa)* extras as classes with numerous active compounds such as amino acids, fatty acids and steroids, which belong to primary metabolites, while cannabinoids, flavonoids, stilbenoids, lignans, terpenoids and alkaloids belongs to secondary metabolites. The main important compounds are the cannabinoids such as Δ9-trans-tetrahidrocanabinol (Δ9-THC), Cannabidiol (CBD), Cannabigerol (CBG) and Cannabinol (CBN), flavonoids (apigenin, luteolin), terpenoids, noncannabinoid phenols and steroids [19].

With respect to *Verbena officinalis* extract, Kubica et al. noted that it contains two phenylpropanoid glycosides (verbascoside and isoverbascoside), 6 phenolic acids (protocatechuic, chlorogenic, vanillic, caffeic, ferulic and rosmarinic acids), iridoids (hastatoside, verbalin and aucubin), flavonoids (luteolin, apigenin, pedalitin, scutellarein) and terpenoids [28]. The main components of *Verbena officinalis* extract are iridoids, phenols or flavonoids.

The *Salvia officinalis* extract is characterized by Pavlic et al. as presenting mainly terpenoids (such as α- and β-thujone, camphor and 1,8-cineole) and a polyphenolic fraction that includes phenolic acids (rosmarinic, ferulic and caffeic acid), flavonoids (luteolin, apigenin and naringenin) and diterpenes (carnosic acid, carnosol, rosmanol, epirosmanol and isorosmanol) [41].

**Figure 2:**
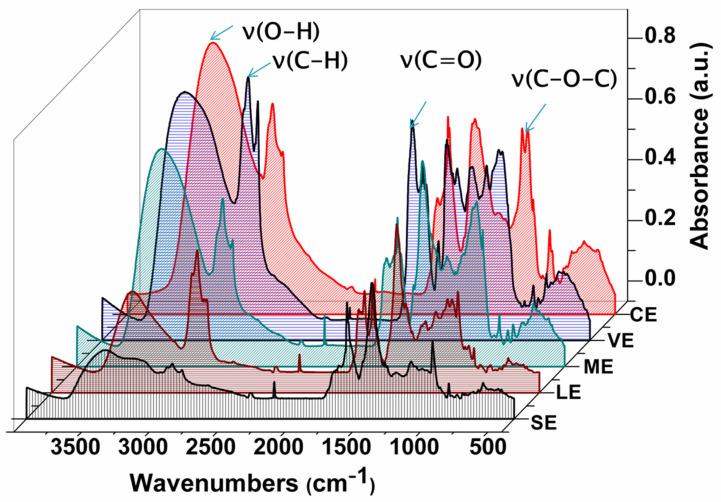
FTIR spectra of the studied plant extracts.

The obtained FTIR spectra of plant extracts were analyzed by defining the registered peaks corresponding to the functional groups (OH-, C-O, C-H) and by indicating the presence of the phenols acid, terpenoids or flavonoids compounds in their structure. This spectrum is described in Figure 2. For all investigated extracts, a broad band that corresponds to –OH groups of alcohols and phenols is registered in the range of 3500 to 3000 cm^−1^ [42]. The highest intensity was observed in hemp plant extracts (*Cannabis sativa* L.). Toward 2970–2800 cm^−1^, we have found peaks with different intensity (2925 and 2853 cm^−1^ for *Lavandula angustifolia*, 2923 and 2850 cm^−1^ for *Mentha piperita*, 2919 and 2851 cm^−1^ for hemp plant (*Cannabis sativa* L.), 2920 and 2850 cm^−1^ for *Verbena officinalis* and 2922 and 2851 cm^−1^ for *Salvia officinalis*). Their presence was connected with the ν(C–H) stretching vibration of symmetric and asymmetric aliphatic groups [43,44,45]. Moreover, the band carbonyl groups ν(C=O) at different peaks (1733 cm^−1^ for CE, 1709 cm^−1^ for VE, 1712 cm^−1^ for ME, 1732 cm^−1^ for LE, 1728 cm^−1^ for SE) indicated the presence of monoterpenes such as thujones, camphor, tannins or flavonoid structure [46,47,48,49]. The different intensity peaks in the range of 1570 to 1230 cm^−1^ were assigned to the alkenes and methyl groups [42]. Furthermore, the large peaks situated in the region 1164–1042 cm^−1^ (1140 and 1091 cm^−1^ for CE; 1169 and 1071 cm^−1^ for VE, 1073 and 1048 cm^−1^ for ME, 1167 and 1076 cm^−1^ for LE) emphasize the presence of ν(C-O-C) stretch vibration and ν(C-O) stretch vibration from flavonoids [44,50,51]. The highest intensity was found in hemp plant extract (*Cannabis sativa* L.). The obtained FTIR spectra identified different classes of compounds.

**Figure 3:**
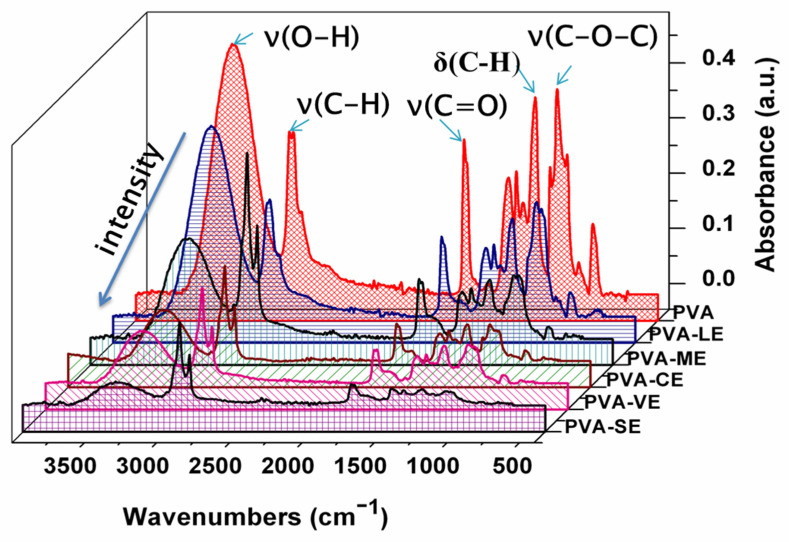
ATR-FTIR spectra of PVA and PVA films loaded with plant extracts.

The spectra of the PVA and PVA loaded with plant extracts are present in the Figure 3.

The analysis shows that the characteristic peaks of poly(vinyl alcohol) and plant extracts have almost similar functional groups, but the intensities of the peaks are different. Thus, the large band between 3500 and 3200 cm^−1^ was attributed to the stretching vibrations ν(O-H) and intermolecular hydrogen bonding vibration [52,53]. Two bands with maxima at 2938 cm^−1^ and 2858 cm^−1^ are the asymmetric and symmetric stretching vibrations ν(C-H) of -CH_2_ groups. At 1733 cm^−1^, it was found the characteristic band of the residual acetate peak and the peaks from the region 1418–1236 cm^−1^ were attributed to the bending vibrations δ(C-H) and δ(O-H) [53,54]. Furthermore, the stretching vibration ν(C-O-C) was located at 1141 cm^−1^ [4,53,55], and the stretching vibration ν(C-O) was found at 1092 cm^−1^ [53]. The presence of plant extracts within the PVA films were evidenced by different features, which proves the existence of intermolecular interactions between them and poly(vinyl alcohol).

In the spectrum of films loaded with *Lavandula angustifolia* extract (PVA-LE), the broad peak corresponding to stretching vibrations ν(O-H), shifts to 3322 cm^−1^ indicated the presence of linalool and terpene-4-ol alcohols in the film [43,56]. The stretching vibration ν(C-H) of methyl from the components terpene-4-ol, linalool and linalyl acetate are manifested by changes in the peaks locations toward lower frequencies such as 2919 cm^−1^ and 2854 cm^−1^ [43].

The presence of carbonyl groups ν(C=O) of the ester components such as linalyl acetate, camphor and lavandulyl acetate [43,46,56] are further confirmed by the band at 1733 cm^−1^ and the stretching vibration ν_(_C-O) of these esters at 1246 cm^−1^ or 1079 cm^−1^. Additionally, the peak associated with ν(C=C) stretching vibrations in components such as linalool, linalyl acetate and terpene-4-ol was found at 1602 cm^−1^ [14,43]. The bands at 858 and 658 cm^−1^ represent the bending vibration δ(C-H) such as that in camphor [46,56]. Moreover, different functional groups are presented in PVA film loaded with *Mentha piperita* extract (PVA-ME). The peak that is attributed to phenols and hydrogen-bonded alcohol was shifted toward 3315 cm^−1^, and those with a higher intensity corresponded to the ν(C–H) stretching vibration of terpenoids such as menthol were shifted toward 2919, 2851 cm^−1^, respectively [44]. Different changes on the stretching vibration band of carbonyl group ν(C=O) characteristic to the menthol esters and terpenes such as camphor, limonene, carvone and pinene were observed [47]. The band is more defined at 1733 cm^−1^, broadening the shoulder of stretching vibration ν(C=C) at 1644.1 cm^−1^. The observed stretch vibration ν(C–O) peaks of alcohol, ethers and ester were founded at 1082 and 1048 cm^−1^, and the peak that went toward to 838 cm^−1^ highlighted the bending vibration δ(C–H) of alkenes [44].

Regarding the PVA film loaded with *hemp* (*Cannabis sativa* L.) extract (PVA-CE), a broad band of phenol ν(O-H) stretch vibration shifts to 3320 cm^−1^ and some sharp peaks attributed to the asymmetric and symmetric ν(C-H) stretches were situated toward 2919 and 2851 cm^−1^ [45]. The spectra indicated the presence of cannabinoids components such as Δ9-trans-tetrahidrocanabinol (Δ9-THC), Cannabidiol (CBD) and flavonoids such as luteolin and apigenin, etc. The typical carbonyl band was at 1733 cm^−1^, and the pronounced absorption peak of the stretching vibration ν(C=C) was defined at 1629 cm^−1^; they were assigned to cannabinoids components [48]. The peaks from the region 1164–1042 cm^−1^, characteristic to flavonoids, ν_(_C-O-C) asymmetric stretch vibration and ν(C-O) stretch vibration were shifted to 1161 cm^−1^ and 1091 cm^−1^, respectively.

As respects the PVA film loaded with *Verbena officinalis* extract (PVA-VE), the band of ν(O-H) stretch vibration attributed to phenols compounds such as phenols acids (protocatechuic, chlorogenic and vanillic) or verbascoside shifted to 3319 cm^−1^ [57]. Peaks of different heights are situated at 2920 cm^−1^ and 2851 cm^−1^, respectively. Their presence was connected with the asymmetric and symmetric stretching vibrations of ν(C–H) of alkanes. The analysis highlighted the common wavenumber for carbonyl structure ν(C=O) bonds at 1732 cm^−1^ from the flavonoid structure such as luteolin, while the stretching vibration ν(C=C) was found at 1631 cm^−1^, respectively, and the stretching vibration ν(C-O) bond shifted to 1086 cm^−1^ from the iridoids structure [50]. Additionally, the bending vibration of δ(C-H) was situated to lower wavenumbers at 948 cm^−1^ [57].

Furthermore, the PVA film loaded with *Salvia officinalis* extract (PVA-SE) relates a broad peak at 3359 cm^−1^ due to the presence of hydroxyl groups, from the phenolic compounds such as rosmarinic acid [11,31]. The asymmetric and symmetric ν(C-H) stretch peaks situated at 2920 cm^−1^ and 2851 cm^−1^ are narrower and better contoured, suggesting the presence of interactions between the structures, whereas the bands within 1461–1366 cm^−1^ were assigned to the symmetric, asymmetric bending of CH_3_ and CH_2_, respectively [49]. The sharp band of stretching vibration of ν_(_C=O) was shifted to 1735 cm^−1^. The authors attributed this peak to camphor [49]. The peaks from the region 1164–1042 cm^−1^ had different heights at 1169 cm^−1^ and 1082 cm^−1^ and were attributed to 1,8-cineole [58]. In addition, the peak positioned at about 722 cm^−1^ was assigned to ν(C-C-O) symmetric ether stretch [49]. In the literature, others authors [59] has also concluded that the sage species contains mainly camphor and 1,8-cineole.

The ATR-FTIR spectrum of PVA films loaded with plant extracts confirms the presence of functional groups such as phenols acids and flavonoids, which are widely reported for their antibacterial property. The compatibility between the poly(vinyl alcohol) and the plant extracts was mainly the result of some hydrogen bond formation. These bonds reveal the probability of antibacterial activity.

### 3.2. UV-Vis Spectroscopy and the Phenols and Flavonoids Content of Extracts

#### 3.2.1. UV-Vis Spectroscopy Analysis 

**Figure 4:**
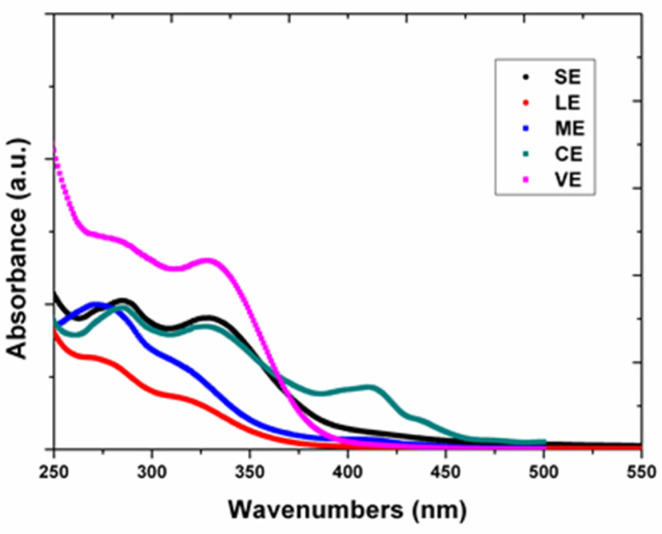
UV-VIS spectra characterization of the plant extracts.

The results of the UV-Vis spectra analysis is presented in Figure 4. The study revealed the peaks of the phenolic compounds and terpenoids for each studied plant extracts in the range of 250–350 nm where according to other researches [42,60,61,62] the identified classes of the compounds generally display certain absorption peaks in the ultraviolet light range.

From the UV-VIS spectra of the extracts, the presence of maximal absorbance for the terpenoids and phenolic compounds such as flavonoids, phenolic acids or tannins were confirmed by the peaks registered at the absorbances of 285 and 331 nm (VE), 286 and 332 nm (CE), 287 and 333 nm (SE), 274 and 312 nm (ME) and 278 and 318 nm (LE), respectively [63]. Furthermore, a maximal absorbance peak was observed in the spectra of *hemp* (*Cannabis sativa* L.) extract at 414 nm specifically for carotenoids such as lutein, β-carotene and lycopene, whose absorption band was recorded in the range of 400 to 500 nm [64,65,66]. The UV-VIS spectrum of *Lavandula angustifolia* extract was characterized by the lowest absorbance throughout the entire wavelength range.

The results of UV-Vis spectroscopy were correlated to the phytochemical analysis spectra based on the identified classes of compounds.

#### 3.2.2. Determination of Total Phenols and Flavonoids Content of Extracts

The secondary metabolites obtained by the plant during growth and reproduction are the phenols. They are produced as a defense against different responses at the infection by pathogens and to the conditions of the environment stress [67]. Flavonoids are compounds responsible for health-promoting properties. A lot of clinical studies have confirmed the safety effects of flavonoids against infections and diseases, such as cardiovascular disorders or cancers [68].

The measurements of the total phenolic content (TPC) and total flavonoid content (TFC) are highlighted in the Table 1. All the studied plant extracts had a generally higher and noticeable total of phenolic contents. The uppermost amounts of the total phenolic and flavonoid contents, respectively, were recorded for *Salvia officinalis* (297.5 mg GAE/g, 46.22 mg QE/g), whereas the minimum ones were observed for *Lavandula angustifolia* (69.61 mg GAE/g, 14.1 mg QE/g). The total phenolic and flavonoid contents decreased in the following order: *Salvia officinalis* (SE)> *Menta piperita* (ME) > *Hemp* (*Cannabis sativa* L.) (CE) > *Verbena officinalis* (VE) > *Lavandula angustifolia* (LE). There is always in the phenolic extract of different plants the presence of different classes of phenols that are exclusively dependent on their solubility in a certain solvent. It is known that the use of ethanol solvent gives a satisfied result to the extraction process for the most of the studied plant species [69]. Our results obtained in the study are consistent with the results of other authors who have measured the TPC of *S. officinalis* at 274.73 mg GAE/g [70], TFC of *hemp* (*Cannabis sativa T*) at 56 mg QE/g [71] or TFC of *Mentha piperita* at 18.20 mg QE/g [72]. Nevertheless, the total of phenolic and flavonoid contents is significantly different among the used species.

### 3.3. Surface Morphology and Surface Roughness Analysis of Films

The application of different polymeric films in medicine (as a wound dressing material) is strongly dependent on biofilm formation due to the adhesion of bacteria at the surface [11,54]. The modifications that occurred in the polymeric films are responsible for preventing the attachment of bacteria to the surface material, which afterwards prevents the extension of the biofilm formation. Some polymeric films known to have antibacterial surfaces are characterized by the mixing of different structures with their composition that cause a disintegration of the bacteria cell membrane [73]. Thus, the surface topography tolerates the bacterial attachment, so a lot of antibiofilm surfaces have been obtained very successfully [74].

**Figure 5:**
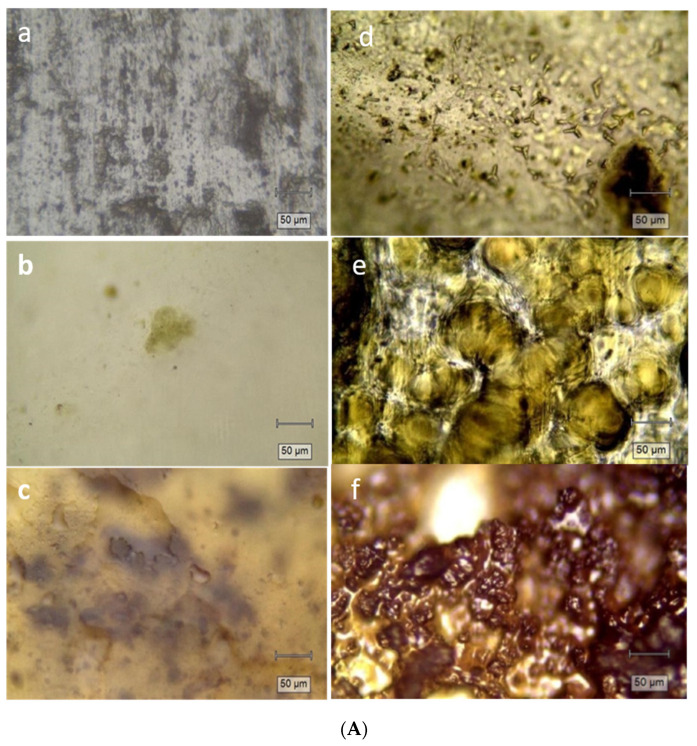

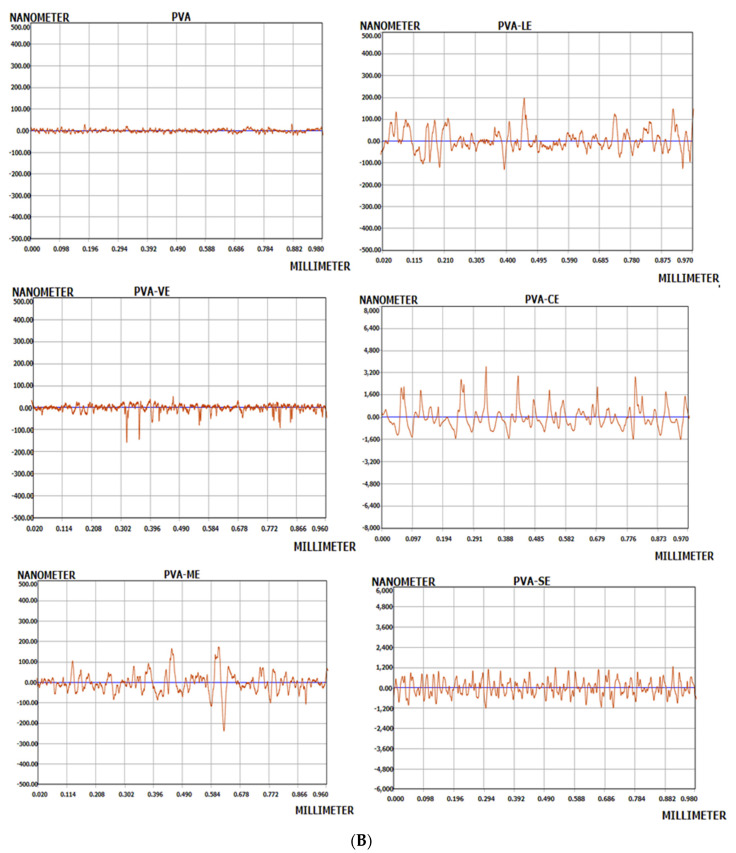
(**A**) Surface morphology analysis with Raman confocal microscope images PVA (a); PVA-VE (b); PVA-ME (c); PVA-LE (d); PVA-CE (e); PVA-SE (f) and (**B**) Profiler roughness measurements.

The arithmetic average of the absolute R_a_ values measured with the profiler of PVA-based films loaded with plants extract was studied. The roughness parameter R_a_ revealed an increase of the surface roughness due to the addition of plant extracts into the pristine PVA film (Table 2). The obtained values of the roughness, R_a_, are from 6.47 nm for pristine PVA [43] to 48.82 nm for PVA-SE. From Table 2, the following trend can be seen: PVA < PVA-VE < PVA-ME < PVA-LE <PVA-CE < PVA-SE. The highest roughness was obtained for the PVA-SE and PVA-CE films. While the Raman confocal microscope image of the pristine surface of PVA (Figure 5) appears almost homogeneous and smooth [75], the films images with plant extracts PVA-VE, PVA-LE, PVA-ME, PVA-CE, or PVA-SE (Figure 5b–f) exhibit relatively rough surface morphology, as observed in the profilometry data of the Figure 5. This is possibly due to the changes in the microstructural characteristics introduced during the mixing between the components. Additionally, the SEM images indicate that the surface of the film without the addition of plant extracts was smooth and homogenous. The incorporation of the tested plant extracts films showed also a relatively rough surface without cracks or any visible pores compared to the control PVA film (Figure S1, Supplementary Information).

In general, it has been found that a higher surface roughness increases the adhesion of bacteria cells [76]. To this end, it is more desirable to obtain a material surface that prevents the bacterial adhesion than to eliminate the microorganisms after they are embedded in the PVA film.

### 3.4. Surface Free Energy Analysis of Films

A first step in the process of development of infection is the attachment of the bacteria to the surface so the physico-chemical properties of the surfaces have an important role in this attachment. Different factors such as hydrophobic interactions, electrostatic attraction and Van der Waals forces initiate the bacterial adhesion at the surface of materials [55]. The measurement of the contact angle is a method that allows the analysis of surface free energy (SFE) and its specific components: polar and dispersive. It was shown in the work of Kocijan [77] that surface free energy besides surface topography is one of the key factors for microorganisms adhesion on surface materials. This technique also determines the hydrophilicity or hydrophobicity of the material and thus helps to evaluate the bacterial potential of the material surface when it is in contact with the biological environment.

In the Table 2, the polar and dispersive components with the values of surface free energy are presented. It is evident that in the case of the films consisting of poly(vinyl alcohol) with plant extracts, the value of surface free energy increased significantly than in that of pristine PVA film. The same tendency was observed in other work, where the additions of the selected plants enhanced the hydrophilicity of the newly obtained materials due to the presence of different components such us -OH groups in their structure [13]. Both polar and dispersive components of the surface free energy were taken into account. The obtained results suggest that in the mixture of PVA film loaded with plant extracts, the polar groups present in the chains of the components were much higher than the dispersive component values and could be redirected towards the inner layer during the film formation [54]. This phenomenon appeared also on other study with polymers based poly(vinyl alcohol) [54].

The mechanism of bacterial adhesion is not so well understood due to several factors, such as different surface properties including roughness, wettability, cell types, protein adsorption, charge and so on. However, most publications have indicated that the materials with a hydrophilic surface are more resistant to the adhesion of bacteria than with a hydrophobic surface [78,79]. Additionally, authors such as McAllister mentioned that the irregularities founded at the polymer surface increase both the biofilm deposition and bacterial adhesion [79]. Regarding the surface free energy, the strain of bacteria and the hydrophobicity surface properties of the polymer must be taken into account. The two bacteria used in our study, *E. coli* and *S. aureus,* expose hydrophobic properties, and most readily they attach to the hydrophobic polymers surface [54].

In order to discuss the results of our work, regarding the surface free energy (SFE), contact angle and the roughness, the obtained results indicate that, in the case of all studied materials, the polar component values were much higher than the dispersive component. This justifies the assumption that our materials with PVA loaded with plant extracts had a primarily hydrophilic surface. Additionally, the studied materials indicate different levels of roughness. This behavior has been also founded in other studies with plant extracts such as *Metha piperita* [80] and lavender [13]. Nevertheless, authors such as Chen et al. [81] mentioned that the strain can expose an affinity to hydrophilic surfaces, which has neutral or positive charges. Thus, the antibacterial activity of the PVA with plant extracts presents a different diameter of inhibition on microorganism growth. Their mechanism of action depends on the chosen plants. Some of them began with the destabilization of the phospholipid structure, keeping on with the interaction of the membrane with the enzymes or proteins and ending with the reduction of the pH gradient across the membrane [82]. Others follow the degradation of the cell wall where the cytoplasmic membrane and the membrane of proteins are damaged, and the content of the cell is leaked [83]. So, considering the obtained results and other information from the literature, it is reasonable to affirm that roughness, contact angle and surface free energy properties have an important role in the antibacterial behavior of PVA films loaded with plant extracts.

### 3.5. Mechanical Properties

**Figure 6:**
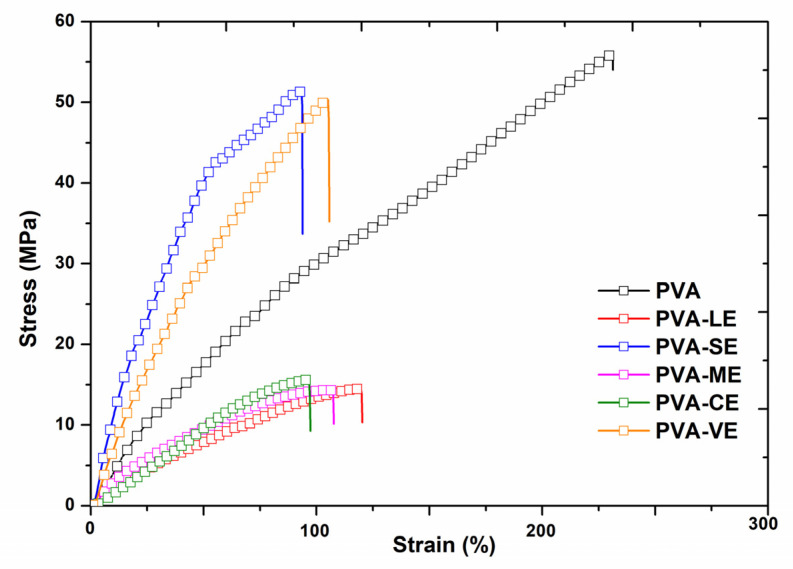
The stress–strain curves of PVA film and PVA loaded with plant extracts.

The mechanical parameters have an important role in the performance of the biomaterial used in a potential application. The mechanical characteristics from Figure 6 show the stress–strain curves of pristine PVA film and of PVA with plant extracts. 

**Figure 7:**
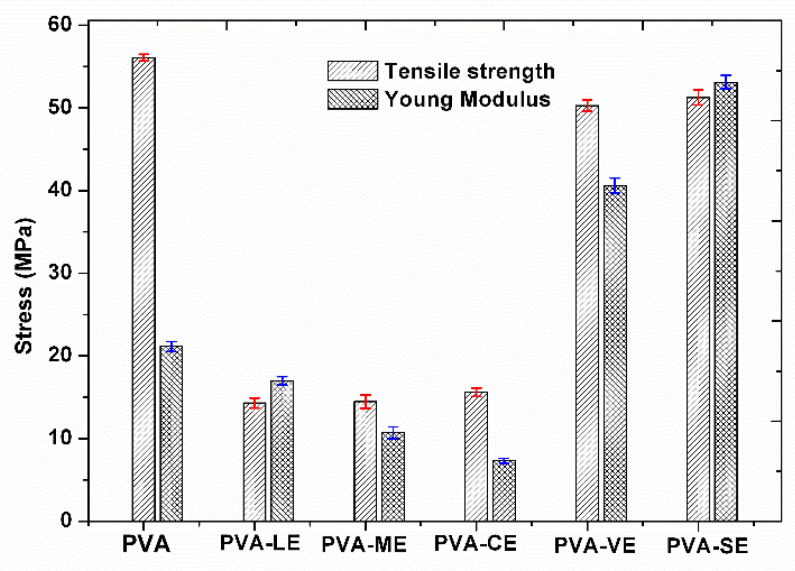
The values of tensile strength and Young modulus of the pristine PVA and PVA with plant extracts.

The obtained films with plant extracts recorded the value of the tensile strength (TS) and the Young’s modulus as being quite diverse. The tensile strength (MPa) defined the capacity of the material to resist rupturing when it is submitted to pressure force. On the other hand, the Young’s modulus defines the elastic inter- and intramolecular forces from the matrix of polymers in order to resist to deformation. The parameters based on the obtained curves are presented in Figure 7.

The value of tensile strength for PVA was 56.07 MPa. This behavior has been also found in PVA films reported in the literature where the value of tensile strength was 53.58 MPa [10]. Furthermore, the films with plant extracts PVA-SE, PVA-VE, PVA-ME, PVA-CE or PVA-LE have tensile strengths 51.26, 50.28, 14.48, 14.31 and 15.61 MPa, respectively. The different value of tensile strength for PVA films with plant extracts may be related to the possibility of a structural rearrangement in the PVA matrix [84]. Some intermolecular interactions such as hydrogen bonding caused by the addition of the extracts are formed [10].

The highest value of tensile strength and the initial modulus was recorded for PVA-SE film and the minimum ones were for PVA-LE and PVA-CE film. The initial modulus decreases from 21.14 MPa at PVA film to 10.72 and 7.31 MPa at PVA-LE and PVA-CE, respectively, meaning that these films deform more easily than the pristine PVA film. This behavior indicates an improvement of the elastomeric characteristics, which may be an advantage for some biomedical applications. Thus, the addition of plant extracts changes the mechanical properties of PVA films.

### 3.6. Antibacterial Activity Analysis

**Figure 8:**
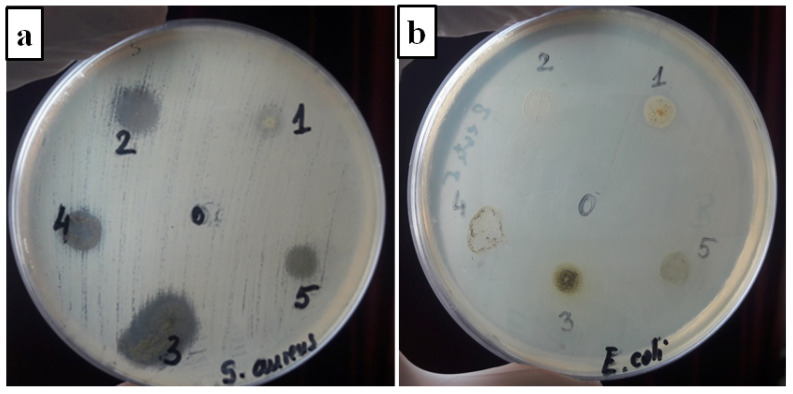
Inhibition zone diameters of PVA and PVA films loaded with plant extracts *S. aureus* (**a**); *E. coli* (**b**). 0, PVA; 1, PVA–LE; 2, PVA–VE; 3, PVA–SE; 4, PVA–CE; 5, PVA–ME.

An amount of different plant extracts (*Lavandula augustifolia, Mentha piperita, Cannabis sativa* L., *Salvia officinalis* and *Verbena officinalis*) was incorporated into PVA after the Soxhlet extraction and the effect on bacterial growth inhibition was evaluated. Thus, using the disk diffusion assay, the inhibition zone around each film was determined due to the penetration of the extract components through the agar. The antibacterial activity was tested again *S. aureus* (ATCC 25922) and *E. coli* (ATCC 25923) bacteria (Figure 8), two of the most predominant species of Gram-positive and Gram-negative strains responsible for severe bloodstream, moderate skin or urinary tract infections [1]. The obtained results are presented in Table 3 and the image in Figure 8.

The inhibitory effect of prepared films varied with different bacteria. The poly(vinyl alcohol) film did not inhibit the growth of each strain, but the films with the plant extracts were very effective in suppressing the growth of the bacteria. The selected films exhibited better bacterial inhibition toward *S. aureus* than *E. coli*. This behavior comes from the differences between the cell wall structure of Gram-positive and Gram-negative bacteria [33]. Gram-negative bacteria such as *E. coli* have a lipopolysaccharide layer on the outer membrane, which prevents the penetration of antibacterial compounds [85,86]. The obtained inhibition zones were within 7–12 mm against *E. coli* bacteria and within 8–20 mm against *S. aureus* strain (Table 3). The highest antibacterial effect again *S. aureus* strain was reported at the films with *Salvia officinalis* (20 mm) extract but for the Gram-negative bacteria *E. coli,* the highest effect was related at the films with *Cannabis sativa* L. (12 mm) extract. The differences in the antibacterial activity of films with plant extracts might be explained by the different active compounds from the structure and also by their diffusivity in the growth media. Thus, the selected plant extracts contain compounds such as phenols, terpenoids and flavonoids, which have different impacts on the growth and metabolism of the microorganisms [17]. The presence of their functional groups (OH-,C-O,C-H) was confirmed by ATR-FTIR analysis (Figure 3).

Thereby, the *Mentha piperita* extract demonstrates its potent antimicrobial activity against a variety of microorganisms because the leaf contains compounds such as phenolic acids (-OH) [87], flavonoids (-CO) or volatile oil as menthol, menthone, menthyl acetate and limonene, which have antibacterial, antifungal and anti-inflammatory properties and interact with the cell membrane of the strains [88,89]. The antibacterial effect of PVA film loaded with *Mentha piperita* extract against *E. coli* and *S. aureus* bacteria was confirmed also by other authors [80], where the degree of antimicrobial activity varies between the cultivars.

A number of studies demonstrated that *Cannabis sativa* L. has been regarded as possessing an antibacterial activity attributed to individual phenolic compounds [13], flavonoids [90] or mainly to cannabinoids oils including CBD [80].The results showed that the film with *Cannabis sativa* L. extract have inhibitory effects on both Gram-positive (*S. aureus*) and Gram-negative bacteria (*E. coli*). This effect was reported also by Wasim, who tested ethanol cannabis extracts against both Gram-positive and Gram-negative strains [91].

Several scientific articles support the antibacterial effects of *Salvia officinalis* extract [33,92,93]. The antibacterial activity is related to phenolic compounds, such as phenolic diterpenoids or phenolic acids, to flavonoids and also others constituents such as 1,8 cineole or camphor [93]. The effects of *Salvia officinalis* on a Gram-negative strain depend on the type of extract used [83]. In contrast with the essential oil of *Salvia officinalis,* which has a significant inhibitory effect on the growth of Gram-negative bacteria, the effect of the ethanol extracts is weak [92,94]. Thus, for our PVA film loaded with *Salvia officinalis* extract, the bacteria growth was found to be very effective against *S. aureus* (20 mm), indicating its greatest effectiveness against this bacterium and one much less for *E. coli* strains (7 mm), as the other authors specified [33,92].

Furthermore, it is known that many antimicrobial compounds from *Lavandula augustifolia* extracts, such as 1,8-cineole, linalool, terpinen-4-ol, α- terpineol or linalyl acetate have been shown to possess antibacterial characteristics against a wide range of microorganisms [1,95,96]. Our film with lavender extract inhibited the growth of both tested bacterial strains, as indicated by the formation of the inhibition zones from 7 mm (against *E. coli*) to 8 mm (against *S. aureus*). The inhibitory effect have been reported also by other authors in their study [13,95], suggesting this important control over microbial growth.

Regarding the PVA film loaded with *Verbena officinalis* extract, it was reported that the antibacterial effect was 13 mm against Gram-positive bacteria in contrast with the Gram-negative strain, where it is showed an inhibition zone of 9 mm. The reason for this might be the presence of compounds such as phenolic acids, flavonoids or phenylpropanoid glycosides: verbascoside and isoverbascoside, known to have antibacterial or antioxidant effect [97]. Several studies were made on *Verbena officinalis* extract showing its potential efficacy on Gram-positive and Gram-negative bacteria [28,97].

These results report that the prepared films with plant extracts are effective in controlling microbial growth and could be a promising material for biomedical applications.

### 3.7. In Vitro Evaluation of Cytotoxicity—MTT Assay of the Films

**Figure 9:**
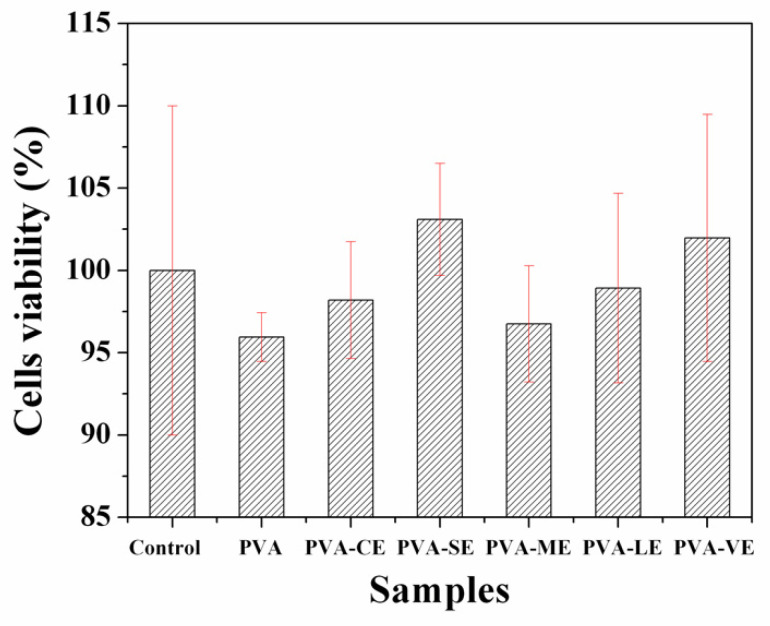
MTT test for PVA and PVA films loaded with plant extracts.

Figure 9 presents the results obtained in the cell viability test of PVA and PVA films loaded with plant extracts tested with fibroblastic cells from the rabbit. Compared to the results of the MTT study of plant extracts, the PVA films loaded with plant extracts are noncytotoxic for the used cells. More than 80% of the cell viability analyzed after 48 h has a very good biocompatibility, after the incubation with the cells (Figure 8). From the data analysis presented in Figure 9, it results that after 48 h of incubation, the cell viability is maintained at the level of growth control. Therefore, the cells are not affected by the potential products released from our materials based on PVA loaded with plant extracts. Thus, the nontoxic effect has been found on other studies with the selected plants [13,71,98,99]. The obtained results certify the biocompatibility of these films.

## 4. Conclusions

In this article, the obtained materials with PVA and different plant extracts were investigated in terms of antibacterial activity. The used plants growing in the flora of Romania were determined by labeling their genus and species. *Lavandula angustifolia, Mentha piperita, hemp* (*Cannabis sativa* L.), *Verbena officinalis* and *Salvia officinalis folium* extracts were obtained and subjected to some studies in order to determine their composition and their possible activity as materials based on poli(vinyl alcohol) used in infection treatment. The total of phenols and flavonoids content and the UV-Vis spectra confirm the presence of different phenolic compounds in the studied plant extracts. The ATR-FTIR and Raman confocal microscope images demonstrated the successful incorporation of plant extracts in the polymer matrix, while the profilometry demonstrated the enhanced surface characteristics for cell growth. Moreover, the ATR-FTIR spectra showed the characteristic components for added plant extracts in the studied materials based on PVA. The hydrophilic nature and roughness parameters of the PVA films loaded with plant extracts were determined by the goniometric and profiler measurements. Diversity in the roughness parameters of *Lavandula angustifolia, Mentha piperita, hemp* (*Cannabis sativa* L.), *Verbena officinalis* and *Salvia officinalis folium* films was caused by the differences in their composition visible also at Raman confocal microscope images.

The results showed that plant extracts conferred significant antibacterial effects to films toward *Staphylococcus aureus* ATCC 25923 and *Escherichia coli* ATCC 25922. The prepared films and plants extracts showed significant antibacterial activities, especially in the films loaded with the *hemp* (*Cannabis sativa* L.) extract against *E. Coli* and in films loaded with *Salvia officinalis folium* against *S. aureus*. The PVA films loaded with plant extracts are noncytotoxic after the test with the used cells. The current study presents scientific news in the field of biomaterials, as there is no literature description of the characterization of *Lavandula angustifolia, Mentha piperita, hemp* (*Cannabis sativa* L.)*, Verbena officinalis* and *Salvia officinalis folium* as PVA film materials loaded with these plant extracts for being used as antibacterial compounds for potential infection treatment. Moreover, the chemical and synthetic antibacterial agents which are known to be harmful to the environment and human health were avoided, and, instead, composite polymeric films were prepared using completely environmentally friendly herbal extracts with PVA polymer, which is frequently used in the biomedical field. By triggering morphological and antibacterial properties of the composites by choosing proper compositions polymer-extract types, it can be allowed to combine unique properties of polymeric materials with completely natural herbal solutions to lead an innovative and eco-friendly approach with potential uses in different pharmaceutical or medical applications.

## Figures and Tables

**Table 1:** The total of phenols and flavonoids content and UV spectra from leaves and flowers of studied plants.

Ethanolic Extract	UV	TPC	TFC
	Absorbance (nm)	(mg GAE/g)	(mg QE/g)
*Lavandula angustifolia*- (Flowers)	278/318	69.61 ± 1.23	14.1 ± 0.56
*Verbena officinalis*-(Leaves)	285/331	102 ± 1.75	30.9 ± 1.24
*Salvia officinalis folium*-(Leaves)	287/333	297.5 ± 0.67	46.22 ± 0.97
Hemp (*Cannabis sativa* L.)-(Leaves)	286/332	76.88 ± 0.98	37.25 ± 1.56
*Mentha piperita*-(Leaves)	274/312	107.05 ± 1.89	26.80 ± 1.78

**Table 2:** Roughness (R_a_), contact angle (**θ_W_),** surface free energy (γ_sv_) and its dispersive (γ^d^_sv_) and polar (γ^p^_sv_) components for PVA and PVA films loaded with plant extracts.

Samples	R_a_ (nm)	θ_W_ (°)	Surface Free Energy (mN/m)		
			γ^p^_sv_ (mN/m)	γ^d^_sv_ (mN/m)	γ_sv_ (mN/m)
PVA	6.47 ± 0.3	65.41 ± 0.7	22.89	16.72	39.61
PVA-LE	23.46 ± 0.5	57.67 ± 0.8	23.10	21.26	44.37
PVA-ME	22.81 ± 0.8	54.53 ± 0.2	27.42	18.56	45.99
PVA-CE	43.95 ± 0.6	44.01 ± 0.6	49.66	6.71	56.38
PVA-VE	12.27 ± 0.2	48.81 ± 0.4	38.07	12.26	50.33
PVA-SE	48.82 ± 0.4	36.76 ± 0.9	56.59	6.15	62.74

**Table 3:** Inhibition zone diameters of PVA and PVA films loaded with plant extracts.

Samples (0.5 mm)	Activity against +/-		Inhibition Zone mm	
	*S. aureus*	*E. coli*	*S. aureus*	*E. coli*
PVA	-	-	0	0
PVA-LE	+	+	8 ± 0.2	7 ± 0.2
PVA-VE	++	+	13 ± 0.3	9 ± 0.1
PVA-SE	++++	+	20 ± 0.1	7 ± 0.3
PVA-CE	++	+++	10 ± 0.2	12 ± 0.4
PVA-ME	++	++	10 ± 0.1	9 ± 0.2

(–) No activity, (+) Weak activity, (++) Good activity, (+++ and ++++) Very good activity.

## Data Availability

The data that support the findings of this study are contained within the article.

## Supplementary Materials

The following supporting information can be downloaded at: https://www.mdpi.com/article/10.3390/ma15072493/s1, Figure S1: PVA (a); PVA-VE (b); PVA-ME (c); PVA-LE (d); PVA-CE (e); PVA-SE (f); Table S1: ATR-FTIR bands present in each films of neat PVA and PVA loaded with plant extracts.
