# Supplementary material for: Poly(vinyl alcohol)/Plant Extracts Films: Preparation, Surface Characterization and Antibacterial Studies against Gram Positive and Gram Negative Bacteria
Source: Materials (Basel). 2022 Mar 28;15(7):2493. doi: 10.3390/ma15072493 (PMC9000143; doi:10.3390/ma15072493)
Supplement: Supplementary file 1 [file materials-15-02493-s001.zip › materials-1611359-supplementary.pdf]

materials

MDPI

Supplementary Materials

Poly(vinyl alcohol)/Plant Extracts Films: Preparation, Surface Characterization and Antibacterial Studies against Gram Positive and Gram Negative Bacteria

ATR-FTIR analysis

The objective of ATR-FTIR analysis was to investigate the registered peaks corresponding to the functional groups (OH-, C-O, C-H) indicating the presence of the phenols acid, terpenoids or flavonoids compounds and to observe the influence of plant extracts on each PVA formulations.

Table S1. ATR-FTIR bands present in each films of neat PVA and PVA loaded with plant extracts.

Wavenumbers			(cm^{-1})			Bond	Type of coumpounds	References
PVA	PVA-LE	PVA-ME	PVA-SE	PVA-CE	PVA-VE			
333	3322	3315	3359	3320	3319	O-H	Hydrogen bonded alcohol, phenols, ν(OH)	[11,42,52–53]
2938	2919	2919	2920	2919	2920	C-H	Stretching $\nu_{as}(CH_3)$, $\nu_{as}(CH_2)$	[3,43–45]
2850	2854	2851	2851	2850	2848	C-H	Stretching $\nu_s(CH_2)$	[4,43–44]
1733	1733	1734	1736	1730	1732	C=O	Stretching ν(C=O)	[4,46–49,56]
1593	1594	1609	1611	1601	1602	C=C	Stretching ν(C=C), aromatic ring bands	[14,48]
1426	1427	1436	1458	1434	1435	C-H	$\delta_s(CH_2)$ and $\delta(CH_3)$, bending, in plane (C-H in CH_2 groups); stretching (C-O-C) of unhydrolyzed acetate groups, in plane (O=H);	[42,53–54]
1373, 1243	1374, 1246	1375, 1247	1375, 1245	1374, 1244	1374, 1247.8	C-H	$\delta_s(CH_3)$, $\delta(CH_2)$,; Bending, in plane (C-H) in CH_2 groups;	[42,53]
1092	1079	1081	1082	1140	1086	C-O	Alcohol, ethers, carboxylic acid, ester, ν(C–O-C)	[44,50–51]
1024	1045	1034	1025	1091	1048	C-O	Alcohol, ethers, carboxylic acid, ester, ν(C–O), bending (O-H)	[4,53,55]
874	848	838.9	851.1	848.6	842.1	C-H	δ (C-H) deformations, Stretching (bending out of plane) (C-H)	[44–45,56–57]

[a]as, asymmetric; s, symmetric; ν, stretching; δ, in-plane bending

Scanning Electron Microscopy (SEM)

The SEM images indicate that the surface of the film without addition of plant extracts was smooth and homogenous. The incorporation of the tested plant extracts films showed a rough surface without cracks or any visible pores compared to the control PVA film.

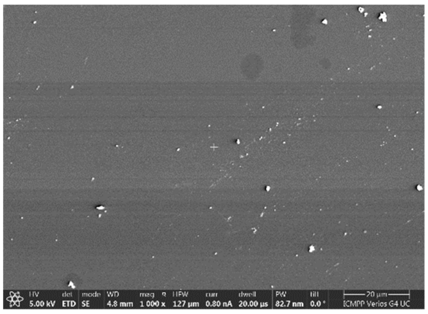

(a)

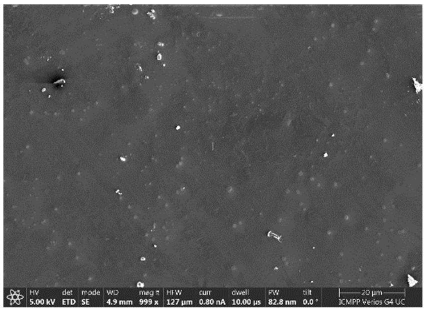

(b)

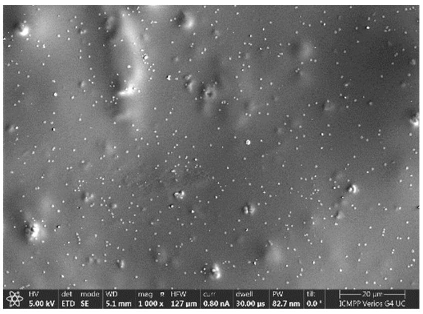

(c)

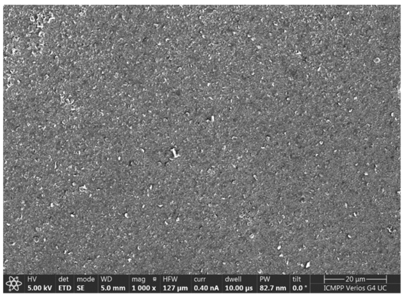

(d)

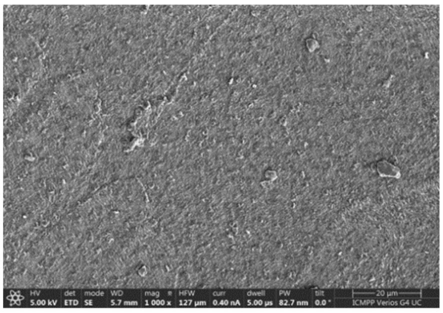

(e)

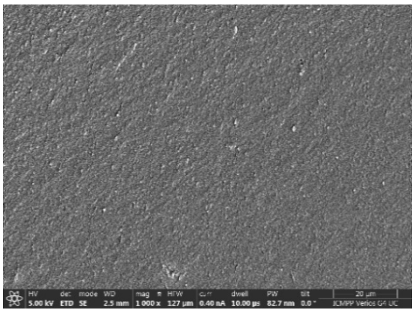

(f)

Figure S1. PVA (**a**); PVA-VE (**b**); PVA-ME (**c**); PVA-LE (**d**); PVA-CE (**e**); PVA-SE (**f**).
